# Mechanical Stress Induces Biotic and Abiotic Stress Responses via a Novel *cis*-Element

**DOI:** 10.1371/journal.pgen.0030172

**Published:** 2007-10-19

**Authors:** Justin W Walley, Sean Coughlan, Matthew E Hudson, Michael F Covington, Roy Kaspi, Gopalan Banu, Stacey L Harmer, Katayoon Dehesh

**Affiliations:** 1 Section of Plant Biology, University of California Davis, Davis, California, United States of America; 2 Agilent Technologies, Wilmington, Delaware, United States of America; 3 Department of Crop Sciences, University Of Illinois, Urbana, Illinois, United States of America; 4 Genomic Medicine, Cleveland Clinic, Cleveland, Ohio, United States of America; The University of North Carolina at Chapel Hill, United States of America

## Abstract

Plants are continuously exposed to a myriad of abiotic and biotic stresses. However, the molecular mechanisms by which these stress signals are perceived and transduced are poorly understood. To begin to identify primary stress signal transduction components, we have focused on genes that respond rapidly (within 5 min) to stress signals. Because it has been hypothesized that detection of physical stress is a mechanism common to mounting a response against a broad range of environmental stresses, we have utilized mechanical wounding as the stress stimulus and performed whole genome microarray analysis of Arabidopsis thaliana leaf tissue. This led to the identification of a number of rapid wound responsive (RWR) genes. Comparison of RWR genes with published abiotic and biotic stress microarray datasets demonstrates a large overlap across a wide range of environmental stresses. Interestingly, RWR genes also exhibit a striking level and pattern of circadian regulation, with induced and repressed genes displaying antiphasic rhythms. Using bioinformatic analysis, we identified a novel motif overrepresented in the promoters of RWR genes, herein designated as the Rapid Stress Response Element (RSRE). We demonstrate in transgenic plants that multimerized RSREs are sufficient to confer a rapid response to both biotic and abiotic stresses in vivo, thereby establishing the functional involvement of this motif in primary transcriptional stress responses. Collectively, our data provide evidence for a novel *cis-*element that is distributed across the promoters of an array of diverse stress-responsive genes, poised to respond immediately and coordinately to stress signals. This structure suggests that plants may have a transcriptional network resembling the general stress signaling pathway in yeast and that the RSRE element may provide the key to this coordinate regulation.

## Author Summary

Plants are sessile organisms constantly challenged by a wide spectrum of biotic and abiotic stresses. These stresses cause considerable losses in crop yields worldwide, while the demand for food and energy is on the rise. Understanding the molecular mechanisms driving stress responses is crucial to devising targeted strategies to engineer stress-tolerant plants. To identify primary stress-responsive genes we examined the transcriptional profile of plants after mechanical wounding, which was used as a brief, inductive stimulus. Comparison of the ensemble of rapid wound response transcripts with published transcript profiles revealed a notable overlap with biotic and abiotic stress-responsive genes. Additional quantitative analyses of selected genes over a wounding time-course enabled classification into two groups: transient and stably expressed. Bioinformatic analysis of rapid wound response gene promoter sequences enabled us to identify a novel DNA motif, designated the Rapid Stress Response Element. This motif is sufficient to confer a rapid response to both biotic and abiotic stresses in vivo, thereby confirming the functional involvement of this motif in the primary transcriptional stress response. The genes we identified may represent initial components of the general stress-response network and may be useful in engineering multi-stress tolerant plants.

## Introduction

Plants are persistently challenged with numerous biotic and abiotic environmental stresses. To cope with environmental stresses plants have evolved phytohormones such as jasmonic acid, salicylic acid, ethylene, and abscisic acid, which are utilized to regulate plant responses to both abiotic and biotic stresses with considerable signaling crosstalk [1,2]. While these phytohormone pathways have been well studied, knowledge of stress perception and initial signaling events, aside from plant pathogen interactions, are less defined. It is known that application of insect oral secretions containing protein fragments of chloroplastic ATP synthase or application of purified oligouronides (OGAs) derived from the plant cell wall are capable of inducing plant defense responses, although a receptor has not yet been identified [3–5]. Additionally, a cellulose synthase (*CESA3*) mutant *cev1* shows enhanced resistance to powdery mildew as a result of constitutive increase in jasmonic acid levels in these plants [6]. This has led to the hypothesis that mechanical disruption of the cell wall may result in stress signaling [3,7]. The perception of cold stress has been hypothesized to be mediated through the detection of changes in membrane fluidity and protein conformation [8–10]. Finally, secondary messengers such as Ca^2+^, reactive oxygen species (ROS), and phosphatidic acid have been implicated in initial signaling cascades in response to both abiotic and biotic stresses [11–17].

One mechanism of response to stress that has been studied extensively in yeast and animals is the general stress response (GSR) (also referred to as the cellular stress response) [18]. The GSR acts in a transient manner in response to a diverse array of stresses. The GSR is initiated in response to strain imposed by environmental forces on macromolecules such as membrane lipids, proteins, and/or DNA. A critical aspect of the GSR, downstream of perception of macromolecular damage, is generation of ROS [19]. Furthermore, key molecular components of the GSR are evolutionarily conserved in all organisms [18].

To better understand plant stress responses, transcript profiling experiments have been successfully employed for many different abiotic and biotic stresses [1,20,21]. One common emerging theme from these experiments is that abiotic and biotic stresses regulate different but overlapping sets of genes [1]. For example, cDNA–amplified fragment length polymorphism analysis of the Avr9- and Cf-9-mediated defense response in tobacco cell culture revealed overlap between race-specific resistance and response to wounding [22,23]. Additionally, partial genome microarray analysis of the *Arabidopsis* wound response revealed that a number of wound-responsive genes encode proteins known to be involved in pathogen defense [24]. Examination of the AtGenExpress abiotic datasets demonstrates that the initial transcriptional abiotic stress response may comprise a core set of multi-stress-responsive genes. The abiotic stress response then becomes stress specific at later time points [25–27]. Finally, recent analysis of the AtGenExpress abiotic and biotic datasets has uncovered ∼200 genes that are expressed in response to a broad range of stresses, which may represent the GSR of *Arabidopsis* [28].

Recently, a shift in stress tolerance engineering has been proposed that transfers the focus from pathway endpoints to factors governing upstream reactions. Focusing on upstream signaling components may enable the engineering of multi-stress tolerance [20,29]. Identification of *cis-*regulatory elements for use in synthetic promoters to confer stress tolerance has also recently been proposed [30,31]. Towards this aim, we have utilized mechanical wounding, as it uniquely confers an instantaneous and synchronous stimulus, to identify primary stress-responsive transcripts. Comparison of the 5 min rapid wound response (RWR) genes we identified with published transcript profiles demonstrated a large overlap with previously identified abiotic and biotic stress-responsive genes. Notably, RWR genes also exhibit a striking level and pattern of circadian regulation. Further investigation via real-time quantitative RT-PCR (RT-qPCR) of a wounding time course revealed genes that are expressed rapidly and transiently as well as rapidly and stably. Two rapidly and transiently expressed genes, *ETHYLENE RESPONSE FACTOR #018* (*ERF#018; AT1G74930*) and *CCR4-ASSOCIATED FACTOR 1* (*CAF1-like; AT3G44260*), were confirmed as wound and biotic stress inducible in vivo using stable transgenic lines expressing transcriptional luciferase fusions. Detailed analysis of the RWR promoters identified a novel *cis*-regulatory element we term the rapid stress response element (RSRE), which is sufficient to confer reporter gene induction in response to abiotic and biotic stress. RWR genes identified in this study may represent initial components of the GSR and be useful in engineering multi-stress tolerance.

## Results/Discussion

### Transcript Profiling Identifies Rapid Wound Response Genes

**Figure 1:**
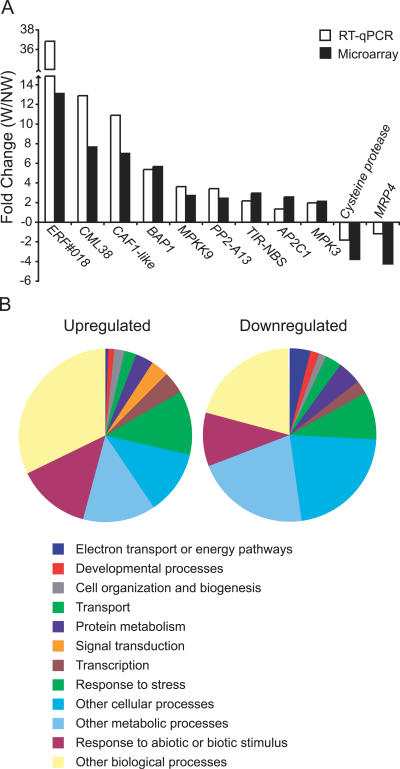
Verification and Functional Classification of the RWR Genes (A) RT-qPCR expression analysis of selected genes normalized to the 60S ribosomal protein L14 (At4g27090) measured in the same samples. The resulting relative expression was then used to calculate fold change upon wounding. Data are means of *n* = 3. (B) Functional classification of RWR genes using GO annotations.

To identify primary stress-responsive transcripts we utilized Agilent microarrays to monitor gene expression changes 5 min after mechanical wounding of *Arabidopsis* rosette leaves. Because of the short duration of our stress treatment we hypothesized that expression changes would be low. In order to accurately detect these changes we utilized three biological replicates of pooled plants per treatment. In addition, two technical replicates, with dye swap of each technical replicate, were performed on each biological replicate. Using this approach, we found that the expression of 162 genes was upregulated and the expression of 44 genes was downregulated at least 2-fold and had a *p*-value ≤ 0.01 five min after mechanical wounding (Table S1). The expression level of selected RWR genes representing a range of high-to-low-fold change was then validated using RT-qPCR. The expression changes determined by RT-qPCR data are in good agreement with the fold change observed by microarray with a spearman rank order correlation coefficient of 0.927 (*p*-value = 0.000) (Figure 1A). RWR genes were then classified according to gene ontology (GO) terms in order to provide insight into their biological function [32]. The two largest defined classes of GO terms involve response to stress or abiotic/biotic stimuli (Figure 1B). It is also of interest to note that genes classified for an involvement in signal transduction were observed in upregulated but not downregulated RWR genes. These data indicate that 5 min of mechanical wounding was sufficient for induction of known stress-responsive genes as well as unknown genes that may play a role in multi-stress responses.

### In Vivo Validation of Rapid Wound Response Genes

**Figure 2:**
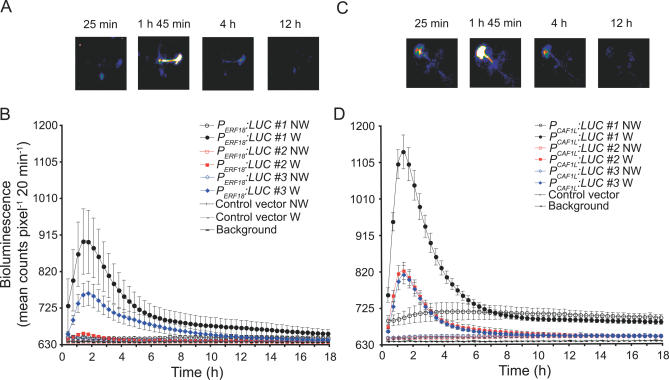
In Vivo Monitoring of RWR Gene Induction Following Wounding (A) Image of an individual *P_ERF18_:LUC #3* transgenic plant over time. (B) Luciferase activity of three independent transgenic lines expressing transcriptional *P_ERF18_:LUC* fusions. Luciferase activity was calculated in wounded leaves (single leaf per plant) and NW leaves (on NW plants). Data are means of *n* = 12 ± SEM. (C) Image of an individual *P_CAF1L_:LUC* transgenic plant over time. (D) Luciferase activity of three independent transgenic lines expressing transcriptional *P_CAF1L_:LUC* fusions. Luciferase activity was calculated in wounded leaves (single leaf per plant) and NW leaves (on NW plants). Data are means of *n* = 18 ± SEM.

We next created stable transgenic lines expressing transcriptional fusions of the *ERF#018* and *CAF1-like* promoters to luciferase to validate in vivo RWR genes and to investigate their temporal expression pattern. For each construct, three independent T2 lines were imaged to control for positional effects of the transgene insertion site. Luciferase activity was then monitored following the wounding of a single leaf per plant to enable the observation of whether the induced activity occurred only locally or also systemically. The wound-induced expression of *P_ERF18_:LUC* occurs rapidly and peaks ∼1 h 45 min after the wound stimulus (Figure 2A and 2B). Additionally, expression of *P_ERF18_:LUC* was observed in the petiole and shoot apex. The expression of *P_CAF1-like_:LUC* was detected rapidly and peaked ∼1 h 25 min surrounding the wound site (Figure 2C and 2D). These data provide in vivo confirmation that RWR genes do respond rapidly to mechanical wounding.

### Expression of RWR Genes over Time

**Figure 3:**
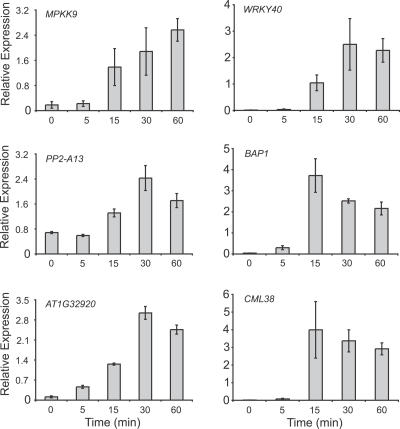
Selected RWR Genes Displaying a Rapid and Stable Expression Pattern Following Mechanical Wounding Total RNA was extracted from 3-wk-old mechanically wounded tissue and subject to RT-qPCR analysis. *AtMPKK9*, *WRKY40*, *AtPP2-A13*, *BAP1*, *AT1G32920*, and *CML38* transcripts were normalized to the 60S ribosomal protein L14 measured in the same samples. Data are means of *n* = 3 ± SEM.

To gain further insight into how the RWR genes may be acting, we performed a RT-qPCR time-course on selected genes. We classified genes as rapidly and stably expressed if 60 min post wounding they remained greater than 2-fold induced. In contrast, we classified genes that had decreased in expression to less than half of maximal expression by 60 min post wounding as rapidly and transiently expressed. Among the rapidly and stably expressed transcripts are genes with either a known or predicted role in stress signal transduction events (Figure 3). *CML38* is a calmodulin-like gene that is predicted to be a sensor of Ca^2+^, a known secondary messenger of stress responses [33]. *MPKK9*, a MAPK signal transduction component, was also identified as rapidly and stably expressed. Additionally, the transcription factor *WRKY40*, which is known to be involved in pathogen defense, was identified [34]. Finally, *BAP1*, a negative regulator of defense responses whose binding of phospholipids is enhanced by calcium, was shown to respond rapidly and stably to wounding [35]. The upregulation of RWR genes in a rapid and stable manner may indicate that these genes play a more prolonged role in response to stress.

**Figure 4:**
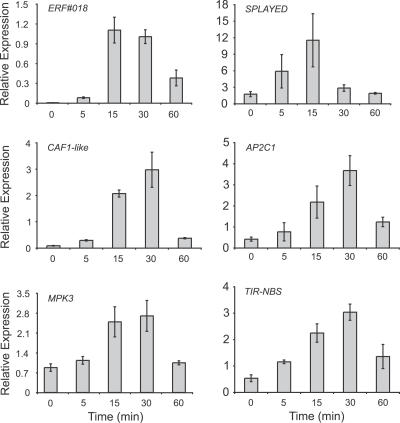
Selected RWR Genes Displaying a Rapid and Transient Expression Following Mechanical Wounding Total RNA was extracted from 3-wk-old mechanically wounded tissue and subject to RT-qPCR analysis. *ERF#018*, *CAF1-like*, *AP2C1*, *MPK3*, and *TIR-NBS* transcripts were normalized to the 60S ribosomal protein L14 while *SPLAYED* transcripts were normalized to *TIP41-like* measured in the same samples. Data are means of *n* = 3 ± SEM.

We also uncovered rapidly and transiently expressed RWR genes with a wide range of functions (Figure 4). One such example is the chromatin remodeling ATPase *SPLAYED* (*SYD*), which peaks 15 min post wounding. Because of the large changes in gene expression following stress it has been hypothesized that chromatin remodeling may be required to allow for stress-induced transcription to occur [36]. Examples of stress-induced changes in histone acetylation state in plants have been described [37,38]. Rapid and transient upregulation of *SYD* suggests that ATP-dependent chromatin remodeling may also take place in order to facilitate downstream stress-induced transcriptional changes.

Genes involved in signal transduction via reversible phosphorylation were also upregulated rapidly and transiently following wounding (Figure 4). One kinase identified was *MPK3*, a MAPK signal transduction component, which has been shown to function in innate immunity and stomatal development [39,40]. *AP2C1*, a PP2C-type phosphatase with a MAPK interaction motif, was also shown to exhibit a rapid and transient expression pattern resulting from wounding [41]. These results indicate that both phosphorylation and dephosphorylation of MAPK signaling components is involved in transduction of initial stress signaling events.

A third process implicated by genes identified in this study is that of mRNA turnover (Figure 4). Specifically, this process is demonstrated by the expression pattern of *CAF1-like*. In yeast, CAF1 has been shown to be a component of the major cytoplasmic deadenylase, which functions to remove the poly(A) tail, thereby initiating mRNA turnover [42]. Additionally, the mouse CAF1 ortholog has been demonstrated to function as a 3′-5′-RNase with a preference for poly(A) substrates [43]. In *Arabidopsis*, RNA processing appears to play a role in response to cold stress [44]. It is also of interest to note the difference in promoter and transcript expression patterns in response to wounding for *ERF#018* and *CAF1-like*. When promoter activity was monitored using transcriptional luciferase fusions, activity peaked ∼1 h 30 min after wounding (Figure 2). In contrast, transcript abundance measured via RT-qPCR peaked 15–30 min after wounding (Figure 4). This discrepancy may be an artifact due to measuring promoter activity via luciferase protein activity, while RT-qPCR assayed transcript levels. An alternative explanation of these results is that mRNA levels of rapidly and transiently expressed genes such as *ERF#018* and *CAF1-like* are controlled post-transcriptionally, possibly via mRNA turnover. In support of this hypothesis, *CAF1-like* mRNA has a half-life of 38 min and has been classified as a gene with an unstable transcript [45]. Furthermore, the mRNA of a second rapid and transient gene, *MPK3,* has a half-life of 43 min and is classified as a gene with an unstable transcript [45].

### RWR Genes Are Regulated by Abiotic and Biotic Stresses

Examination of the RWR genes reveals a large number of known genes involved in abiotic and biotic stress responses (Table S1). Among the upregulated RWR genes were genes involved in ethylene signaling including *ACC synthase 6* (*ACS6*) as well as 15 of the 122 ethylene response factors (ERFs) in *Arabidopsis*, which have been shown to be involved in the response to both biotic and abiotic stresses [46]. The transcriptional activators *CBF1* (*DREB1B*), *CBF2* (*DREB1C*), and *CBF3* (*DREB1A*), which confer tolerance to cold and drought, were also among the RWR genes [47–50]. Additional RWR genes known to confer tolerance to a range of abiotic stresses include *STZ* (*ZAT10*) and *ZAT12* [51,52]. RWR genes also include genes with a known function in response to biotic stress. Examples include *BAP1*, a negative regulator of defense responses [35]. *MPK3* and *FLS2* which function in response to pathogen-associated molecular patterns in the *Arabidopsis* innate immune response [39,53]. The transcription factor *TGA3* which regulates *pathogenesis-related* (PR) genes and is required for basal pathogen resistance was also among the RWR genes [54]. Finally, *ERD15* regulates not only cold and drought tolerance but also resistance to the bacterial necrotroph Erwinia carotovora subsp. *carotovora* [55].

**Figure 5:**
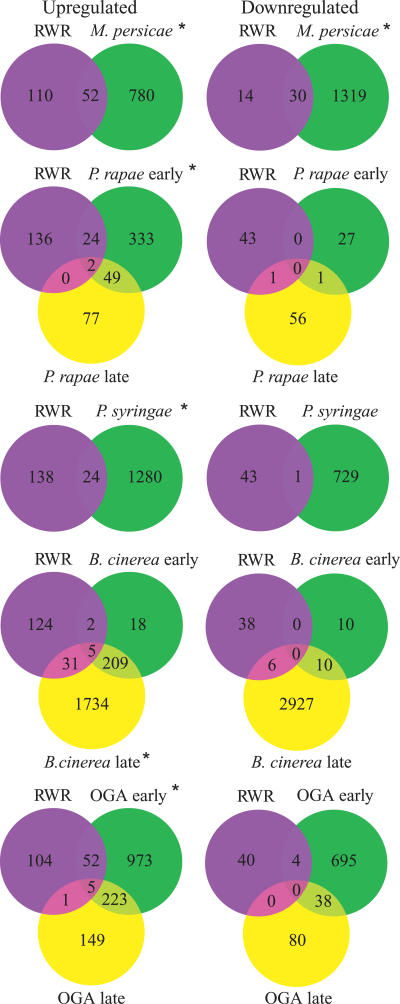
Comparison of RWR Genes with the Transcript Profile of Other Environmental Stimuli An asterisk denotes a statistically significant overrepresentation of RWR genes in the transcript profile of the indicated stress (*p* < 0.0001). *Arabidopsis* plants were challenged with M. persicae for 48 h and 72 h; P. rapae early for 5 h; P. rapae late and P. syringae for 12 h and 24 h; *B cinerea* early and late for 18 h and 48 h, respectively; and OGA early and late for 1 h and 3 h, respectively [59–61].

The abundance of RWR genes with known abiotic and biotic stress tolerance functions led us to examine the role of wounding as a general stress perception mechanism on a global level. For this analysis, we compared the overlap in gene lists between the RWR genes and published transcript profiles for a number of stress conditions. The statistical significance of the observed overlap in transcript profiles was then analyzed using empirical permutation tests [56]. We first compared RWR genes with published abiotic microarrays and found a strong overlap (unpublished data), which is in agreement with work recently published by Kilian et al. [25]. For example, 49% of upregulated RWR genes have been previously shown to be upregulated upon cold treatment [57,58]. Additionally, four of the nine genes (*At1g27730*, *At5g51190*, *At5g47230*, and *At5g04340*) found by Kilian et al. [25] to be upregulated by 30 min of cold, drought, UV-B, salt, osmotic stress treatment, and wounding we discovered to be upregulated within 5 min of wounding. We next compared the RWR transcript profile with published transcript profiles of plants challenged with different biotic stresses [59–61]. For upregulated datasets there was a statistically significant overrepresentation of RWR genes in the transcript profile of all biotic stresses tested (Figure 5). Furthermore, the overrepresentation of RWR genes occurred only at early time points of P. rapae and OGA stressed plants. These data indicate that perception of mechanical stress may play a central role in the perception and initial response to a wide range of environmental stresses.

### RWR Genes Respond to Biological Elicitors

**Figure 6:**
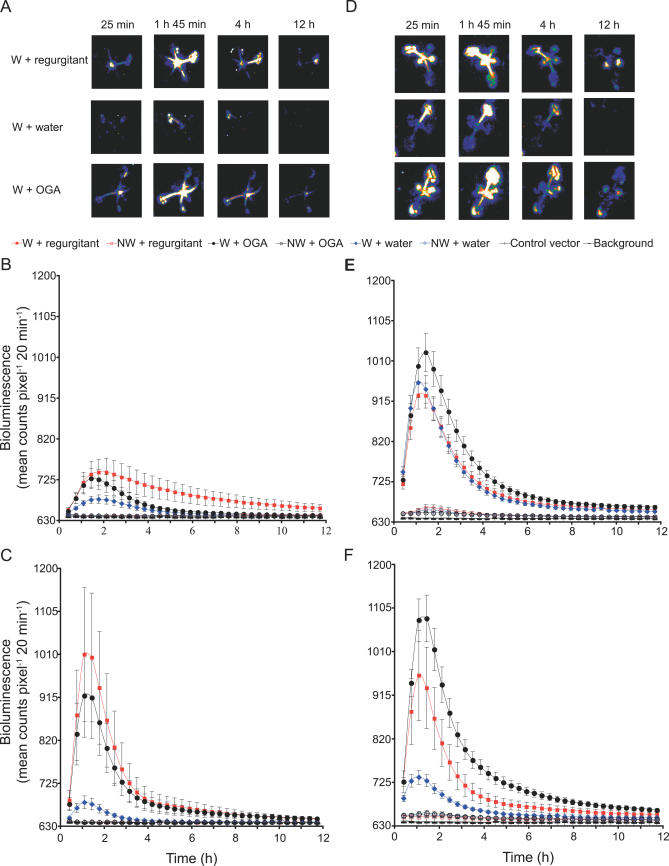
In Vivo Monitoring of RWR Gene Induction upon Addition of Biotic Elicitors One leaf per plant was either NW or wounded (W) and then treated with oligouronides (OGA), IR, or double-distilled H_2_O. (A) Image of an individual *P_ERF18_:LUC #3* transgenic plant over time. (B) Local expression in the treated leaves of *P_ERF18_:LUC #3* transgenic plants. Data are means of *n* = 9 ± SEM. (C) Systemic expression of *P_ERF18_:LUC #3* plants monitored in the shoot apex. Data are means of *n* = 9 ± SEM. (D) Image of an individual *P_CAF1L_:LUC #2* transgenic plant over time. (E) Local expression in the treated leaves of *P_CAF1L_:LUC #2* transgenic plants. Data are means of *n* = 12 ± SEM. (F) Systemic expression of *P_CAF1L_:LUC #2* plants monitored in the shoot apex. Data are means of *n* = 12 ± SEM.

Various biological compounds are known to elicit stress-signaling networks. Due to the overlap between RWR genes and biotic stresses we tested whether the RWR genes *ERF#018* and *CAF1-like* respond to the biological elicitors OGA and insect regurgitant (IR) as well as cabbage looper (Trichoplusia ni) feeding. We first tested whether *P_ERF18_:LUC* or *P_CAF1-like_:LUC* activity was induced by cabbage looper feeding. Indeed, cabbage looper feeding did result in enhanced luciferase activity, which verified that biological stress does induce *ERF#018* and *CAF1-like* (Videos S1 and S2). We therefore proceeded to test induction resulting from OGA and IR treatment. When OGA, IR, or H_2_O were added to a nonwounded (NW) leaf, no induction of *P_ERF18_:LUC* or *P_CAF1-like_:LUC* activity was observed (Figure 6A–6F). The lack of induction is likely due to the application method we used (single droplet per plant) rather than an actual lack of response to the elicitors. When transcript profiling was performed on liquid cultured 10-d-old plants incubated with 50 μg ml^−1^ OGAs, *CAF1-like* was shown to be induced 1 h after addition of OGA to the media [61].

In *P_ERF18_:LUC*-expressing plants, addition of OGA and IR to the wound site resulted in a significantly greater (*p* < 0.05) induction of luciferase activity than addition of H_2_O in both local and systemic tissue (Figure 6B and 6C, respectively). In contrast, addition of OGA or IR to the wound site did not result in a significant difference (*p* > 0.05) in *P_CAF1-like_:LUC* activity compared to addition of H_2_O to the wound site in local tissue (Figure 6E). However, a significantly greater induction, compared to H_2_O, in *P_CAF1-like_:LUC* activity resulted from addition of OGA or IR to the wound site in systemic tissue (Figure 6F).

There are a number of common second messengers downstream of mechanical wounding, cabbage looper feeding, and OGA treatment that may signal for the observed induction of RWR genes. One such secondary messenger is Ca^2+^, which increases in intracellular concentration rapidly following wounding as well as OGA treatment [3,16,62,63]. ROS are another secondary messenger that have been shown to increase in response to chewing insects, wounding, and OGA treatment [3,12]. Furthermore, while OGAs do not move systemically, ROS do accumulate systemically following wounding. This increase in ROS is likely through OGAs released by systemically induced polygalacturonase [14,16,64–66]. Finally, OGA, chewing insects, and wounding may all have a common mechanism of perception resulting in similarly induced secondary messengers. Both chewing insects and wounding have a physical effect on the plasma membrane. The perception of OGA has also been hypothesized to be a result of its physical effect on the plasma membrane, rather than through an actual receptor [3,67].

### Circadian Regulation of the RWR Genes

**Figure 7:**
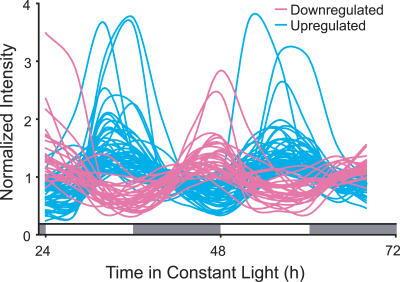
Circadian Regulation of RWR Genes These data are comprised of circadian-regulated RWR genes. Upregulated RWR genes (blue) peak at dusk while downregulated RWR genes (pink) peak at dawn. Plants were entrained in light/dark cycles for 7 d and then released into constant light. Samples were collected every 4 h after plants were moved to constant light.

The circadian clock has been shown to regulate a number of environmentally regulated genes [68]. Additionally, cold-induced expression of RWR genes *ZAT12*, *CBF1*, *CBF2*, and *CBF3* was recently reported to be gated by the circadian clock [69]. These findings led us to examine globally whether RWR genes are under circadian regulation. Towards this aim, we compared the RWR genes with genes recently identified as circadian regulated [56]. Surprisingly, not only were RWR genes rhythmically expressed but the upregulated and downregulated genes also showed unexpected phase distributions (Figure 7). Forty-two percent of RWR upregulated genes are expressed at subjective dusk while 81% of downregulated RWR genes peak at subjective dawn (*p* ≤ 0.0001). The circadian regulation of RWR genes may provide a mechanism to anticipate stresses caused by daily environmental changes.

### Identification of a Novel Stress-Responsive *cis*-Regulatory Element

**Figure 8:**
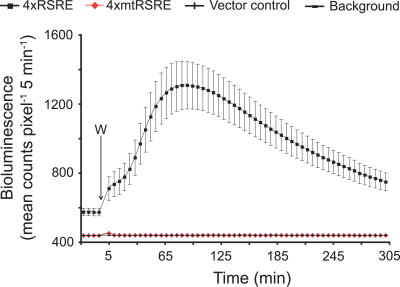
The RSRE Confers Wound-Induced Reporter Gene Expression Independent T1 *4xRSRE:LUC* and *4xmtRSRE:LUC* lines were mechanically wounded. Luciferase activity was then monitored in the wounded leaf. Data are means of *n* = 24 ± SEM.

To begin dissecting the molecular mechanism underpinning the rapid stress response, we examined the promoters of the RWR genes for novel *cis*-regulatory elements. We identified the six-nucleotide repeat, CGCGTT, which we are terming the Rapid Stress Response Element (RSRE), as significantly overrepresented (58 hits in 47 of the 162 upregulated promoters) in the promoters of upregulated RWR genes. To determine whether the RSRE is sufficient alone to confer stress-responsive transcription, we used luciferase reporter constructs. Four tandem repeats of the RSRE and its consensus flanking sequence were separated by six nucleotides and cloned upstream of the minimal promoter region of the nopaline synthase (NOS) gene and modified luciferase coding region (*4xRSRE:LUC*). Additionally, to verify that the RSRE was the region conferring stress responsiveness, we mutated three of the six nucleotides in the RSRE (*4xmtRSRE:LUC*). The wound-induced expression of these constructs was then tested in 24 independent T1 plants to control for differences in expression resulting from the site of transgene insertion. All 24 *4xRSRE:LUC* transgenic plants exhibited wound-induced luciferase expression (Figure 8). Furthermore, luciferase activity increased immediately following wounding and peaked ∼80 min post wounding. Conversely, no *4xmtRSRE:LUC* transgenic plant exhibited luciferase activity before or after wounding. These data demonstrate that the RSRE is sufficient to confer a rapid response to stress.

**Figure 9:**
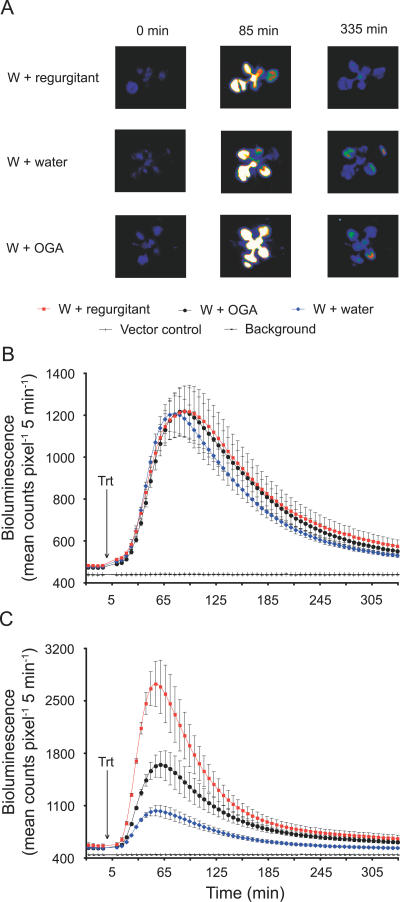
The RSRE Responds to Biotic Elicitors (A) Image of an individual *4xRSRE:LUC* transgenic plant over time that was wounded and treated with OGA, IR, or H_2_O. (B) Local expression in the treated leaves of *4xRSRE:LUC* transgenic plants. (C) Systemic expression of *4xRSRE:LUC* in the shoot apex. Data are means of 12 ± SEM.

Both abiotic and biotic stresses appear to share common signaling components with the RWR. We were therefore interested in whether the RSRE confers a rapid response to a range of stresses. To enable accurate quantification of the stress response we used a homozygous T3 *4xRSRE:LUC* line. Because RWR genes respond to both OGAs and IR (Figures 3 and 7), we tested the expression of *4xRSRE:LUC* following treatment with these biotic elicitors. In the local leaf where the wound site was treated with OGA or IR, no induction over that of H_2_O treatment was observed (Figure 9A and 9B). Notably, in systemic tissues, a statistically significant (*p* < 0.05) synergistic enhancement of luciferase activity was detected in OGA- and IR-treated plants when compared to H_2_O treatment (Figure 9A and 9C). To further demonstrate that the RSRE responds to biotic stress, *4xRSRE:LUC* plants were challenged with cabbage loopers and B. cinerea. When *4xRSRE:LUC* plants were exposed to both forms of biotic stress, luciferase activity was induced, whereas no activity was observed in vector control lines (Videos S3 and S4). Additionally, in B. cinerea–infected plants, a low level of transient luciferase activity was first observed in the inoculated leaf. Luciferase activity was then observed at a greater level in systemic tissues (Video S4). These data clearly demonstrate that the RSRE responds to biotic stress.

**Figure 10:**
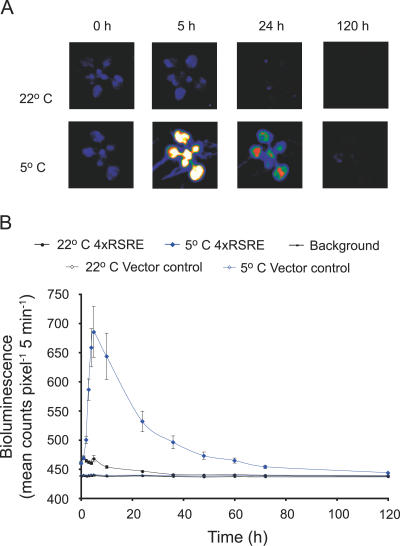
The RSRE Confers Cold-Induced Reporter Gene Expression (A) Image of an individual *4xRSRE:LUC* transgenic plant incubated at 5 °C or 22 °C over time. (B) Luciferase activity of *4xRSRE:LUC* plants. Data are means of 16 ± SEM.

While the RSRE responds to the abiotic stress of mechanical wounding, we wished to further demonstrate the role of the RSRE in response to abiotic stress. Towards this aim, we exposed *4xRSRE:LUC* expressing plants to 5 °C. Plants were then removed from cold treatment at the indicated time for imaging. Additionally, control *4xRSRE:LUC* plants were also kept at 22 °C in equivalent light conditions and moved similarly to cold-treated plants to ensure that transfer to the imaging chamber did not result in induced luciferase activity. Induction of luciferase activity was observed after ∼2 h of cold treatment (Figure 10A and 10B). Furthermore, luciferase activity peaked after 5 h of cold treatment and then decreased towards basal expression levels. Notably, *4xRSRE:LUC* expression was also observed in the roots of cold-treated plants. To ensure that the *4xRSRE:LUC* plants were still competent to express luciferase, they were mechanically wounded after 120 h of cold treatment. Both cold- and 22 °C-treated *4xRSRE:LUC* plants exhibited luciferase induction following wounding (unpublished data). These data demonstrate that the RSRE is cold responsive and that initial signaling events leading to a cold response dampen even in continuous exposure to cold.

The rapid and transient response of the RSRE to multiple stress conditions is reminiscent of the yeast GSR promoter element STRE (stress response element; AGGGG) [18]. The STRE is responsible for rapid induction following various treatments such as heat, nitrogen starvation, low external pH, osmotic, and oxidative stress [70–73]. Furthermore, even in the presence of continuous stress exposure, STRE-mediated gene induction dampens over time. An increase in unsaturated fatty acids upon stress appears to be responsible for the transient nature of STRE-mediated induction [74,75]. When plants are exposed to abiotic and biotic stresses (cold and P. syringae, respectively), there is an increase in unsaturated fatty acids [9,76]. Upon cold treatment, acyl-lipid desaturases are the enzymes that most efficiently introduce double bonds in membrane lipids, which results in the increased level of unsaturation [9]. Similar to cold treatment, two of the RWR upregulated genes are acyl-lipid desaturases (*ADS1* and *ADS2*), which may increase the unsaturation of membranes upon wounding. It is therefore tempting to speculate that, as with the STRE in yeast, the increase in unsaturated membrane lipids resulting from both abiotic and biotic stresses may mediate the transient induction of RSRE-driven reporter gene expression.

### Conclusions

We have shown that 5 min of mechanical stress is a sufficient amount of time for the plant to perceive the stress and mount a robust transcriptional response. The rapid transcriptional response to mechanical wounding shares a large overlap with both abiotic and biotic stresses and may therefore represent the initial GSR of *Arabidopsis*. In support of this view, the RWR upregulated genes comprised 25% of the genes identified as potential GSR genes via analysis of the AtGenExpress abiotic and biotic stress datasets [28]. Additionally, in mammalian cells, physical stress to membranes during osmotic and UV radiation stress result in the nonspecific clustering of growth factor receptor tyrosine kinases and cytokine receptors [18,77]. A similar nonspecific clustering of receptors during mechanical wounding and other environmental stresses may underlie the GSR of plants. We also show that the RWR genes are circadian regulated with consolidated phases of peak expression. Circadian regulation of RWR genes, which likely encompass initial components of the GSR, may enable plants to anticipate daily environmental changes and mount a general defense against these changes. Finally, we identified a *cis*-regulatory element (RSRE) overrepresented in the promoters of RWR genes. The RSRE confers a rapid and transient response similar to the yeast GSR promoter element (STRE) and is a novel GSR *cis*-regulatory element in plants.

Since the RWR genes likely represent initial components of the GSR, they provide a valuable resource of candidate genes for engineering of multi-stress resistance. Similarly, the RSRE, which responds rapidly and transiently to abiotic and biotic stresses, may prove useful as a synthetic element for engineering of multi-stress tolerance. Finally, use of the RSRE in yeast one-hybrid and the *4xRSRE:LUC* line for mutant screens should help elucidate the upstream mechanisms of stress perception and initial signal transduction.

## Materials and Methods

### Plant growth conditions and treatment.

*Arabidopsis* (Arabidopsis thaliana) ecotype Columbia-0 plants were grown in a 16 h light/8 h dark photoperiod at 22 °C. All experiments were conducted on 3-wk-old soil-grown plants unless otherwise noted. All rosette leaves (unless otherwise noted) were mechanically wounded one to two times with a hemostat (resulting in ∼20% of the leaf being damaged). Mechanical wounding was performed 4–6 h after dawn.

### Cloning.

For cloning of *P_ERF18_:LUC*, the 1.4 kb upstream of the translation start site of *ERF#018* (At1g74930) was PCR amplified using primers listed in Table S2. For cloning of *P_CAF1-like_:LUC*, the 2-kb upstream region of the translation start site of *CAF1-like* (AT3G44260) was PCR amplified using primers listed in Table S2. The PCR amplicons were cloned into the pENTR/D-TOPO vector and subcloned into the Gateway destination vector pBGWL7 [78] by an LR reaction (Invitrogen, Carlsbad, CA). *4xRSRE*:LUC (5′-cataaCGCGTTtttagatatcataaCGCGTTtttaggatccataaCGCGTTtttatctagaataaCGCGTTtttac-3′) and *4xmtRSRE:LUC* (5′-cataaCATGCTtttagatatcataaCATGCTtttaggatccataaCATGCTtttatctagaataaCATGCTtttac-3′) constructs were created by cloning into the SacI/XhoI sites of pATM-Nos [79]. Transformations were performed into Columbia-0 plants by floral dip using Agrobacterium tumefaciens strain GV3101 [80].

### Arabidopsis thaliana oligoarray and preparation of labeled targets for hybridization.

The *Arabidopsis* 2.0 oliogoarray chip containing 60-mer oligos and representing a total of 21,500 probes (TAIR ATH1 v4.0) was obtained from Agilent (G4137A; Wilmington, Delaware). Total RNA from leaf tissue was isolated using TRIzol reagent (Invitrogen) following the manufacturer's suggested protocol. Prior to hybridizations, the quality and quantity of the total RNA sample was confirmed by running 10-ng samples on an Agilent bioanalyzer (RNA chip), and by using a spectrophotometer. The oligoarray hybridization experiments utilized three biological replicates of pooled plants (∼40 plants per pool). Each biological replicate was comprised of two technical replicates with dye reversal. Total RNA (500 ng) was used as a template for cRNA production and cyanine dyes were incorporated using the Agilent low RNA input linear amp kit (Agilent). Normal yields from 500 ng of total RNA input using a 4-h in vitro transcription were 15 μg cRNA (15 pmol cyanine dye incorporated/μg cRNA).

### Array hybridization and scanning.

One microgram of labeled cRNA (cy3- and cy5-labeled sample) was diluted to 175 μl and defragmented at 60 °C for 30 min following the Agilent hybridization protocols (Agilent). Defragmented samples were diluted to 500 μl (30% formamide final concentration) and hybridized for 20 h at 40 °C. Arrays were washed and dried and scanned on an Agilent G2565BA microarray scanner [81]. The raw TIFF images were analyzed using the Agilent Feature Extraction software v. 8.1 using the recommended default settings.

### Microarray data analysis procedures.

The intensities of Cy3- and Cy5-labeled probes were normalized by comparing signal intensities of housekeeping genes (positive controls) for both dyes and using the determined ratio as a correction factor for differences in labeling efficiencies (Agilent Feature Extraction v. 8.1 software). The genes that had valid signal in all six replicates were exported to Rosetta Resolver software and analyzed according to the manufacturer's instructions (Seattle, Washington). The normalized values were used to calculate the ratio of channel intensities (Cy5/Cy3), which were then log_10_ transformed. The transformed ratios were plotted in a scatter plot (cy5/cy3) A ±1.7-fold increase or decrease in signal intensity (*p*-chance value, 0.01) “Signature” Features (>1.5-fold deviation from the median, *p*-chance value 0.01) was exported into Microsoft Excel for further analysis. In Excel, data were sorted and genes with a fold-change ≥ 2.0 were selected as differentially regulated. Additionally, differentially expressed genes were binned based on signal intensity.

### RT-qPCR.

RT-qPCR analysis was conducted based on Yamagishi et al. [82] Total RNA was isolated by TRIzol extraction (Life Technologies, Grand Island, NY) and treated with DNase, MseI, and DdeI to control for DNA contamination. One microgram of RNA was reverse transcribed using Superscript III (Invitrogen, Carlsbad, California). PCRs were conducted using 12.5 μl of SYBR Green mix (40 mM Tris HCL pH 8.4, 100 mM KCl, 6 mM MgCl_2_, 8 % glycerol, 20 nM fluorescein, 0.4× SYBR Green [Molecular Probes, Eugene, Oregon], 100-fold dilution of BSA [New England Biolabs, Beverly, MA], and 1.6 mM dNTP), 2 μl of a 30-fold dilution of the RT reaction, 0.6 U iTaq DNA Polymerase (Bio-Rad Laboratories, Hercules, CA), 9.38 μl H2O, and 0.24 μM of each primer. Reactions were carried out on a Bio-Rad iCycler iQ multicolor real-time detection system (Bio-Rad Laboratories) using a two-step reaction condition (extension temperature was primer specific but was typically ∼60 °C), followed by a melt curve encompassing 80 steps of 0.5 °C from 60 °C to 100 °C. Gene-specific primers were designed using Beacon Designer software (Premier Biosoft Palo Alto, CA) and are listed in Table S2. Primary data analysis was performed with Bio-Rad iCycler iQ software. The Bio-Rad gene expression macro version 1.1 software (Bio-Rad Laboratories) was used to calculate relative RNA levels normalized to an internal control [83–85]. The 60S ribosomal protein L14 (At4g27090) described in [82] or the TIP41-like gene (At4g34270) described in [86] was used as an internal control.

### Comparison of transcript profiles.

For this analysis, we compared the overlap in gene lists between the RWR genes and published transcript profiles determining circadian-regulated genes as well as abiotic and biotic stress-responsive genes. The statistical significance of the observed overlap in transcript profiles was then analyzed using a recently described empirical permutation test [56] based on sampled randomization testing [87] with a *p*-value cutoff of *p* < 0.0001.

### Luciferase imaging.

For luciferase imaging, 10–14-d-old plants grown on plates containing 1× Murashige and Skoog basal salt mixture (Sigma) were utilized. Plants were sprayed with 2.5 mM luciferin (Promega, Madison, WI) in 0.001% Triton X-100 ∼16–20 h prior to treatment. Mechanical wounding was performed on a single leaf per plant. For elicitor application, 5 μl of 30 mg/ml OGA, 1μl of cabbage looper (Trichoplusia ni) regurgitant, or 5 μl of sterile _dd_H_2_O was applied to a single leaf. For cold treatment, plants were placed in a 5 °C chamber under low light. Control plants were kept at 22 °C in equivalent light conditions and handled similarly to cold-treated plants to ensure that transfer to the imaging chamber did not result in induced luciferase activity. Plants were then removed from cold treatment for imaging and returned to either cold or 22 °C. Five microliters of Botrytis cinerea isolate KB2 in 1/2× grape juice was inoculated on a single leaf at a concentration of 500 spores/μl [88,89]. Plants were imaged using an Andor DU434-BV CCD camera (Andor Technology, South Windsor, CT). For image acquisition, *P_ERF18_:LUC* or *P_CAF1-like_:LUC* plants were exposed for 20 min while *4xRSRE:LUC* plants were exposed for 5 min. Luciferase activity was quantified for a defined area (leaf, shoot apex, or whole plant) as mean counts pixel^−1^ exposure time^−1^ using Andor Solis image analysis software (Andor Technology, South Windsor, CT). For statistical analysis of treatment effects, the area under the curve was calculated and compared by Kruskal-Wallis one way ANOVA on ranks, with pairwise multiple comparisons (Student-Newman-Keuls Method), using Sigma Stat v3.5 (San Jose, CA).

### Oligouronide preparation.

A 1% (w/v) solution of citrus pectin (Sigma-Aldrich) in 0.5 M HCl was refluxed for 3 h at a rolling boil. Following cooling, the sample was neutralized with NaOH, decolorized using activated charcoal, and dialyzed against water using 6,000 molecular weight tubing. The dialyzed sample was then lyophilized [90].

### Detection of statistically significant promoter motifs.

Promoter sequences were defined as a fixed distance (2 kb for the purpose of motif detection) upstream of the annotated translation start codon, as described in Hudson and Quail [90]. An enhanced enumerative motif recognition algorithm was developed based on that described by Hudson and Quail [90]. The enhanced method does not require exact motif matching, and permits degenerate bases to occur in any number at any position through the search motif by searching for and enumerating all possible permutations of bases within the specified motif size limits including wildcard (*N*) bases. Significant associations between promoter gene lists and all permutations of motifs are then detected by comparison of per-promoter motif abundance between the promoters of the target coregulated gene list and the promoters of all genes in the *Arabidopsis* genome. The per-promoter binomial test described [91] is used to rank motifs in order of significance, with the exception that a more efficient factorial handling algorithm was used to perform the binomial test on every motif present in the list of coregulated promoters, rendering the preliminary chi-square filtering step unnecessary. Finally, motifs with related sequence are aligned and automatically clustered for output based on nucleotide-level identity. Programs were written in the Perl programming language utilizing the GMP Multiple Precision Arithmetic Library [92]. The *Arabidopsis* promoter motif search program is available for use via a web interface at http://stan.cropsci.uiuc.edu/tools.php. Source code is available from MEH on request.

## Supporting Information

Table S1 List of Upregulated and Downregulated RWR Genes (88 KB XLS)

Table S2 Primers Used for RT-qPCR and Cloning (38 KB XLS)

Video S1 Movie of a *P_ERF18_:LUC*-Expressing Plant Challenged with Cabbage Loopers for 18 h (595 KB MOV)

Video S2 Movie of *P_CAF1-like_:LUC*-Expressing Plants Challenged with Cabbage Loopers for 18 h (1.4 MB MOV)

Video S3 Movie of *4xRSRE:LUC*-Expressing Plants Challenged with Cabbage Loopers for 16 h (659 KB MOV)

Video S4 Movie of a *4xRSRE:LUC*-Expressing Plant Inoculated with B. cinere
Induction of *4xRSRE:LUC* activity appears first in the inoculated leaf and subsequently spreads systemically. Images were taken for 5 min every 30 min. This movie consists of images 24–48 h post inoculation.
(3.0 MB MOV)

### Accession Numbers

The data discussed in this publication have been deposited in the National Center for Biotechnology Information (NCBI) Gene Expression Omnibus (GEO, http://www.ncbi.nlm.nih.gov/geo/) and are accessible through GEO Series accession number GSE8740.

## Abbreviations

ERF: ethylene response factor
GO: gene ontology
GSR: general stress response
IR: insect regurgitant
NW: nonwounded
OGA: oligouronide
ROS: reactive oxygen species
RSRE: rapid stress response element
RT-qPCR: real-time quantitative RT-PCR
RWR: rapid wound response
SEM: standard error of the mean
STRE: stress response element
W: wounded
